# Impact of Non-dietary Nutrients Intake on Misclassification in the Estimation of Nutrient Intake in Epidemiologic Study

**DOI:** 10.2188/jea.16.193

**Published:** 2006-09-04

**Authors:** Mikiko Ogata, Shinichi Kuriyama, Yuki Sato, Taichi Shimazu, Naoki Nakaya, Kaori Ohmori, Atsushi Hozawa, Ichiro Tsuji

**Affiliations:** 1 The Division of Epidemiology, Department of Public Health and Forensic Medicine, Tohoku University Graduate School of Medicine.

**Keywords:** Calcium, Dietary, misclassification, non-dietary intake, Thiamine, Ascorbic Acid, Vitamin E

## Abstract

**BACKGROUND:**

Few previous epidemiologic studies have evaluated the effects of non-dietary nutrient intake, such as supplements, over the counter (OTC) drugs, and prescription drugs containing vitamins or minerals, in examining the relationship between dietary factors and health outcomes.

**METHODS:**

To examine the influence of the non-dietary intake of vitamins and calcium on the estimation of nutrient intake, we conducted a cross-sectional study with 1,168 community-dwelling Japanese subjects aged 70 years or older in 2002. The subjects were asked to bring their non-dietary nutrient sources to the examining site. The dietary and non-dietary intakes of vitamins B_1_, C, E and calcium were obtained and the subjects were grouped into quartiles according to their dietary intake and their dietary plus non-dietary intake. The degree of agreement between these two classifications was examined to estimate the degree of misclassification.

**RESULTS:**

Among the subjects who were classified into the highest intake category for vitamin E with dietary intake plus non-dietary nutrient intake, 34.2 % were misclassified into lower category with dietary intake alone. Similarly, intake of vitamin B_1_, vitamin C and calcium were misclassified 28.8%, 18.8 %, 6.2 %, respectively.

**CONCLUSIONS:**

Our data suggest that estimation of vitamin intake from dietary sources alone would yield a maximum misclassification of one-third, which would lead to misleading conclusions being drawn from epidemiologic studies. In contrast, the degree of misclassification for calcium may be relatively small.

In recent years, there have been inconsistent results from epidemiologic studies of the relationship between the intake of fruits and vegetables and health outcomes. For example, eight prospective cohort studies were conducted during a 12 year period from 1994 through 2005 to determine the relationship between intake of fruit and vegetables and the incidence of colorectal cancer,^1^^-^^8^ but the results were inconsistent. Two of these studies showed inverse associations,^1^^,^^2^ while the other six studies showed no such relationship.^3^^-^^8^ One of the hypothesized mechanisms underlying the relationship, if any, has been the role of micronutrients, such as vitamin C, in fruit and vegetables, which La Vecchia et al^9^ described. However, only two studies^5^^,^^7^ considered the use of non-dietary sources of nutrients, and they only treated use of vitamin supplements as control variables in their multivariate models.

According to the results of The National Nutrition Survey in Japan in 2001,^10^ 20.5 % of the people regularly took vitamin and mineral supplements. In the United States, the Third National Health and Nutrition Examination Survey (NHANES III)^11^ reported that 54.4 % of Americans aged 70 years or older and 57.0 % of non-Hispanic Caucasian women took such supplements. Thus, quite a large proportion of the population is taking vitamin and mineral supplements, which may influence nutrient intake substantially.

Block et al^12^ examined sources of vitamin C and E intake on the basis of US National Surveys. Vitamin supplements were the top contributors to the intake of these nutrients and represented 45.6 % of total vitamin E intake and 27.5 % of vitamin C intake. They concluded that failure to consider supplements in the estimation of vitamin intake would result in considerable misclassification in nutrient intake estimation.^12^ Messerer et al^13^ examined the effect of inclusion of dietary supplement use on the validity of micronutrient estimates, and reported that the validity of questionnaire-based micronutrient intake estimates is increased by including dietary supplement use. Although there are a few studies from western countries mentioned above, no study, to our knowledge, has clarified the degree of misclassification in nutrient intake estimation yielded by non-dietary nutrient sources in Japan, where the way of supplement use much differs from western countries.

We therefore investigated the impact of non-dietary vitamin and calcium intake on misclassification in the nutrient intake estimation among Japanese elderly people. In our study, the degree of misclassification was clarified for the first time in Japan. Our study results may be useful in epidemiologic studies of the relationship between dietary factors and health outcomes.

## METHODS

### Subjects and Recruitment

The Tsurugaya Project was a community-based Comprehensive Geriatric Assessment (CGA) of elderly Japanese individuals living in Tsurugaya district, a suburban area of Sendai city in northern Japan, in July and August 2002. At the time of the study, there were 2,730 people aged 70 years or older living in the Tsurugaya district.^14^^-^^16^ We sent invitation letters to all of these people asking them to participate in the health survey. Of those invited, 1,198 participated in the survey and 1,178 (43.2 %) gave written informed consent to be included in the analysis. The protocol for this study was approved by the Institutional Review Board of Tohoku University Graduate School of Medicine.

Supplements were defined as nutrients supplied in addition to those obtained in the diet, excluding over the counter (OTC) drugs and prescription drugs. These supplements included health “pep-up” drinks. All subjects were asked by letter and telephone to bring all supplements, OTC drugs, and prescription drugs they had taken almost every day over the past month to the examination site, along with their packaging. Ten subjects who failed to bring vitamin and mineral supplements, OTC drugs, and prescription drugs to the examination site were excluded from the analysis. Therefore, 1,168 subjects were enrolled in the analysis.

### Examination of Non-dietary Nutrient Intake

The non-dietary vitamin and calcium intake included vitamin and mineral supplements, OTC drugs, and prescription drugs. All subjects were asked to bring all vitamin and mineral supplements, OTC drugs, and prescription drugs to the examination site. We did not have enough time to ask the subjects in detail about their non-dietary nutrient intake. Therefore, one trained pharmacist photocopied the labels of the containers of the non-dietary nutrients and then transcribed the name, content, daily dosage and manufacturer’s name from each supplement, OTC drug, or prescription drug container. A few containers of the supplements had no description of the volume or the content for vitamin B_1_ (six supplements), vitamin C (six supplements), vitamin E (11 supplements), and calcium (seven supplements). Therefore, we considered these supplements did not include these nutrients.

Multi-vitamin was defined as non-dietary vitamin intake containing vitamin A, B, C, and E, as well as provitamin A. The daily intake of vitamins and calcium was estimated from the recommended daily intake shown on the container of the vitamin and mineral supplements, and the daily dosage of OTC drugs and prescription drugs.

### Examination of Dietary Nutrient Intake

The examination was conducted by an in-person interview for all subjects. The short-version food frequency questionnaire (FFQ) developed by Sasaki et al^17^ was used for evaluation of the intake of foods and nutrients, including 55 food items over the past month. Food items were grouped into categories that included cereals; meat; fish; vegetables; fruits; seaweeds; milk products; soybean products; egg; pickles; sweats; seasonings; beverages; alcoholic beverages. The answer to the question regarding the frequency and the approximate amount of intake was evaluated according to 7 possible frequency categories in decreasing order from “twice a day or more” to “never”. However, beverages and rice and miso soup (Japanese traditional soup) were evaluated according to 8 possible frequency categories in decreasing order from “at least 4 cups a day” to “never”, 9 possible frequency categories from “8 bowls or cups or more” to “never”, respectively. The Standard Tables of Food Composition in Japan (4th Edition) were used to analyze nutrient intake,^18^ and vitamin E was estimated by reference to specific tables of food composition.^19^ Fortified foods were not considered in the present FFQ.

Sasaki et al^17^ reported on the validity of the original FFQ. The questionnaire comprised 110 food items and presented choices for the approximate volume of intake. In a validity study of the original FFQ, the FFQ was compared with 3-day dietary records among 47 women (aged 38 to 69 years) with mild hyperlipidemia. Pearson’s correlation coefficient for the intake, estimated on the basis of the FFQ of the subjects and the intake estimated on the basis of the diet record, was 0.46 for thiamin (vitamin B_1_), 0.45 for vitamin C and 0.49 for calcium. The validity was not examined for vitamin E. At present, there is no report on the validity of the short version of the FFQ.

### Statistical Analysis

Vitamin B_1_, vitamin C, vitamin E, and calcium were selected and analyzed because these are used most commonly (Table 1). Although vitamin B_6_ and vitamin B_12_ are also common, we chose vitamin B_1_ as a representative of the vitamin B group for simplicity.

**Table 1.** The number of subjects taking non-dietary vitamins and calcium in the Tsurugaya Project (n=1168), Japan, 2002.

Type of nutrient	Subjects taking non-dietary nutrients*	

	No.	%
Vitamin A	18	1.6
ProVitamin A	13	1.1
Vitamin E	147	12.6
Vitamin K	7	0.6
Vitamin B_1_	116	9.9
Vitamin B_2_	47	4.0
Vitamin B_6_	90	7.7
Vitamin B_12_	85	7.3
Niacin	16	1.4
Other vitamin B group^†^	18	1.5
Vitamin C	87	7.4
Multivitamin	7	0.6
Total vitamins	354	30.3
Calcium in men	14	1.2
Calcium in women	70	6.0

* : Non-dietary nutrient sources included vitamin and calcium supplements, over the counter drugs, and prescription drugs.

† : Other vitamin B group included pantotenic acid, folic acid and biotin.

The scatter plots were presented, and the Pearson’s correlation coefficient was calculated to analyze the relationship between vitamins and calcium dietary intake alone and dietary intake plus non-dietary vitamin and calcium intake.

The subjects were allocated to quartiles according to dietary intake plus non-dietary nutrient intake. Similarly, the subjects were classified into quartiles according to the vitamin and calcium intake estimated from dietary intake alone. The degree of misclassification was determined on the basis of the degree of agreement between the estimate from dietary intake plus non-dietary nutrient intake, and the estimate from dietary intake alone.

Paired t-test was used for comparison of dietary vitamin B_1_, vitamin C, vitamin E, and calcium intake alone, and dietary intake plus non-dietary nutrient intake. All statistical tests were two-sided. A *p*-value of < 0.05 was accepted as statistically significant. All analyses were performed with SAS^®^ software (version 8.2; SAS Institute, Inc, Cary, NC).

## RESULTS

The mean age of the subjects was 75.7 years (standard deviation (SD), 4.7). For non-dietary nutrient users, vitamin and mineral supplement plus OTC drug users had a mean age of 74.5 years (SD, 4.6), and prescription drugs users had a mean age of 76.0 years (SD, 4.8).

### Non-dietary Vitamin C, Vitamin E or Calcium Intake

Of 1,168 subjects, 354 subjects (30.3 %) reported taking non-dietary vitamins (Table 1). The 354 users of non-dietary nutrients comprised 102 men and 252 women. Non-dietary vitamin E was used most frequently, followed in order by vitamin B_1_, vitamin B_6_, vitamin C, vitamin B_12_, calcium in women, vitamin B_2_, vitamin A, niacin, provitamin A, and other members of the vitamin B group (Table 1).

### Comparison of Dietary Intake Alone and Dietary Intake Plus Non-dietary Intake

The mean intakes of vitamin B_1_, vitamin C, vitamin E and calcium estimated from the FFQ were 0.75 mg (SD, 0.22), 103.9 mg (SD, 44.9), 5.8 mg (SD, 1.8) and 654.5 mg (SD, 239.6), respectively. The mean intake of dietary vitamins and calcium and the dietary plus non-dietary nutrient intake were compared (Table 2). The mean dietary plus non-dietary intakes of vitamin B_1_, vitamin C, vitamin E, and calcium were significantly higher than the dietary intakes alone (p < 0.001). For vitamin B_1_, the mean estimate with dietary intake plus non-dietary nutrient intake was 11.2-fold higher than the mean estimate with dietary intake alone. Similarly, for vitamin E and vitamin C, the mean estimates with dietary intake plus non-dietary nutrient intake were 6.7-fold and 1.5-fold higher than the mean estimate with dietary intake alone. There was a modest but significant difference in calcium intake.

**Table 2.** Comparison of dietary intake alone and dietary intake plus non-dietary nutrient intake in the Tsurugaya Project (n=1168), Japan, 2002.

	Nutrient intake from dietary sources alone	Nutrient intake from dietary + Non- dietary sources*	p value^‡^
	Mean intake (mg) (SD^†^)	Mean intake (mg) (SD^†^)	
Vitamin B_1_	0.75 (0.22)	8.39 (27.05)	< 0.001
Vitamin C	103.9 (44.9)	153.6 (262.8)	< 0.001
Vitamin E	5.8 (1.8)^§^	39.1 (114.0)	< 0.001
Calcium	654.5 (239.6)	674.4 (268.0)	< 0.001

* : Non-dietary nutrient sources included vitamin and calcium supplements, over the counter drugs, and prescription drugs.

† : Standard Deviation

‡ : Paired t-test was used

§ : mg *α*-TE (alpha-tocopherol equivalent)

**Figure 1.**
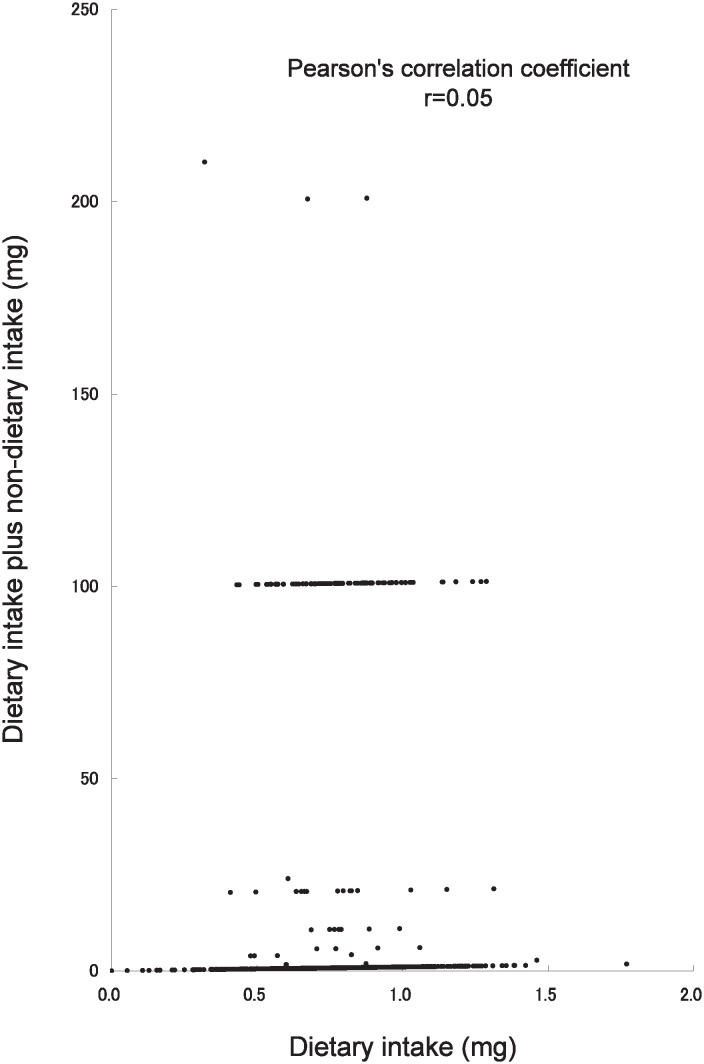
Scatter plot of vitamin B_1_ dietary intake alone and dietary plus non-dietary intake in the Tsurugaya Project (n=1168), Japan, 2002.

**Figure 2.**
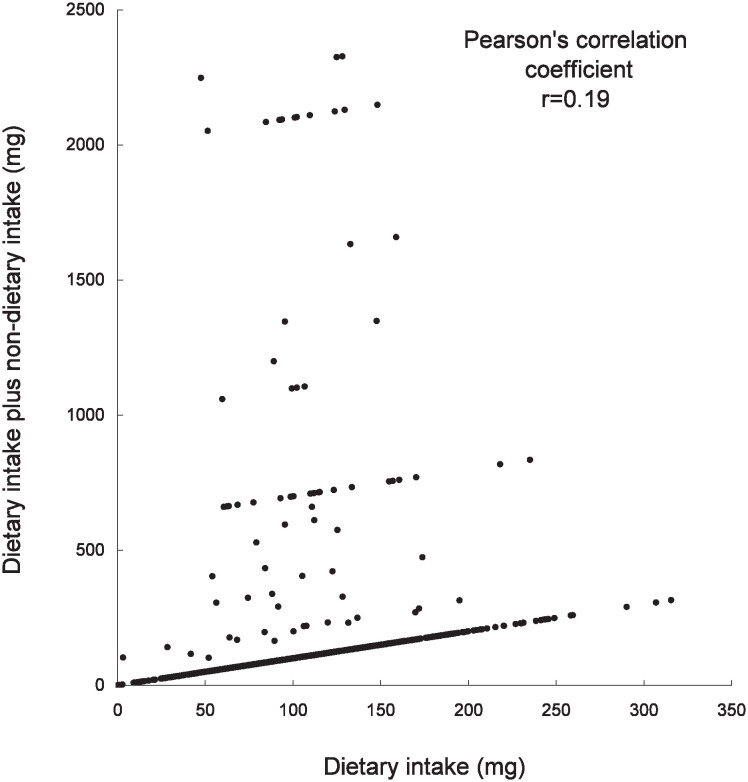
Scatter plot of vitamin C dietary intake alone and dietary plus non-dietary intake in the Tsurugaya Project (n=1168), Japan, 2002.

**Figure 3.**
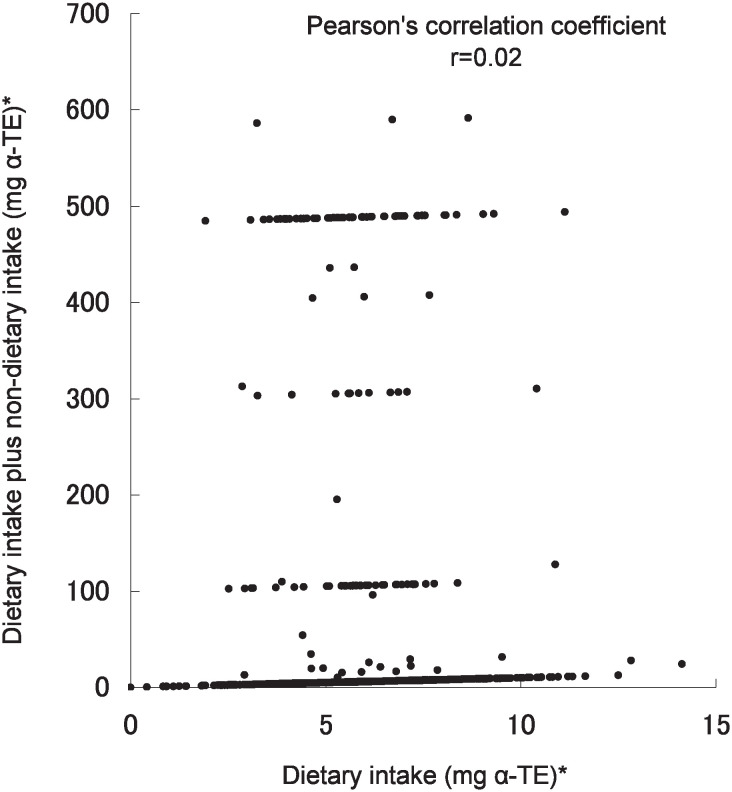
Scatter plot of vitamin E dietary intake alone and dietary plus non-dietary intake in the Tsurugaya Project (n=1168), Japan, 2002. * : alpha-tocopherol equivalent

**Figure 4.**
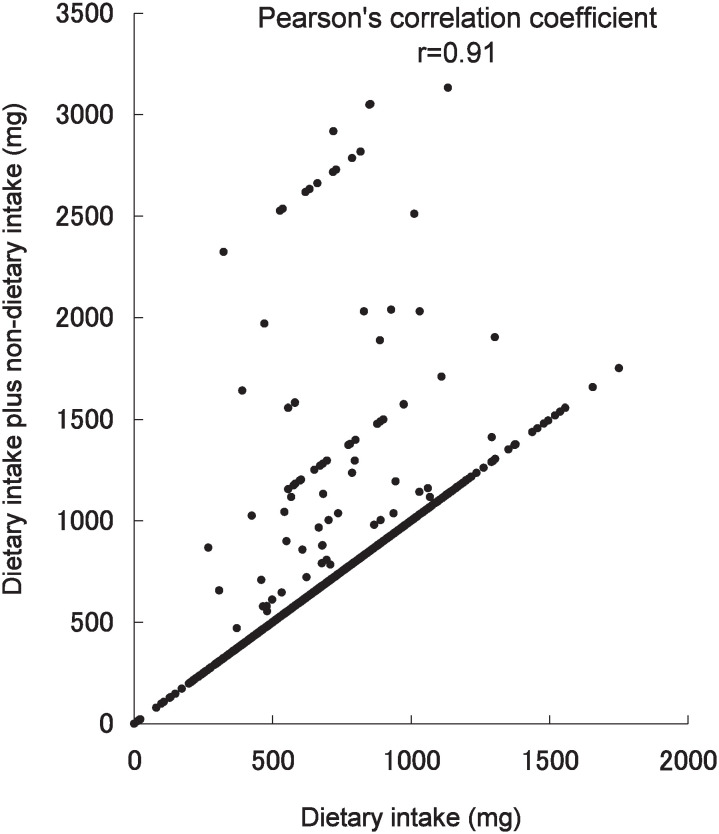
Scatter plot of calcium dietary intake alone and dietary plus non-dietary intake in the Tsurugaya Project (n=1168), Japan, 2002.

The Pearson’s correlation coefficients for vitamins and calcium dietary intake alone and dietary plus non-dietary intake were 0.05 for vitamin B_1_, 0.19 for vitamin C, 0.02 for vitamin E, and 0.91 for calcium. Thus the correlation coefficient for calcium was the highest (Figures 1-4). 

For vitamin B_1_, among the 292 subjects who were classified as quartile 1 (the highest intake) on the basis of the estimate from dietary intake plus non-dietary nutrient intake, 28.8% were misclassified as quartile 2, 3, or 4 in the estimate based on dietary intake alone (Table 3). Among the subjects classified as quartile 2 in the estimate based on dietary intake plus non-dietary nutrient intake, 28.8% were misclassified as quartile 1 in the estimate based on dietary intake alone. Similarly, 16.8% in quartile 3 and 8.2% in quartile 4 (the lowest) were misclassified (Table 3). The overall degree of agreement between these two classifications was 79.4% for vitamin B_1_.

**Table 3.** Distribution of vitamin B_1_ intake estimation by quartiles of dietary intake alone and dietary plus non-dietary intake in the Tsurugaya Project (n=1168),* Japan, 2002.

Quartiles of dietary intake alone	Intake (mg)		Quartiles of the estimate from dietary intake + Non-dietary intake(mg)^†^				Total

			1 (highest)	2	3	4 (lowest)	
			≥0.9351	0.7681 – 0.9350	0.6372 – 0.7680	<0.6372	
1 (highest)	≥0.8809	n(%)	208 (71.2)	84 (28.8)	0 (0.0)	0 (0.0)	292 (100)
2	0.7470 – 0.8808	n(%)	35 (12.0)	208 (71.2)	49 (16.8)	0 (0.0)	292 (100)
3	0.6249 – 0.7469	n(%)	25 (8.6)	0 (0.0)	243 (83.2)	24 (8.2)	292 (100)
4 (lowest)	<0.6249	n(%)	24 (8.2)	0 (0.0)	0 (0.0)	268 (91.8)	292 (100)

Total		n(%)	292 (100)	292 (100)	292 (100)	292 (100)	1168 (100)

* : Subjects were grouped into quartiles according to their dietary intake and their dietary plus non-dietary intake.

† : Non-dietary nutrient sources included vitamin supplement, over the counter drugs, and prescription drugs.

For vitamin C, among the 292 subjects who were classified as quartile 1 (the highest intake) on the basis of the estimate from dietary intake plus non-dietary nutrient intake, 18.8 % were misclassified as quartile 2, 3, or 4 in the estimate from dietary intake alone (Table 4). Among the subjects classified as quartile 2 in the estimate from dietary intake plus non-dietary nutrient intake, 19.1% were misclassified as quartile 1 or 4 in the estimate from dietary intake alone. Similarly, 12.0 % in quartile 3 and 5.5 % in quartile 4 (the lowest) were misclassified (Table 4). The overall degree of agreement between these two classifications was 86.1% for vitamin C. Thus, overall, 13.9 % of subjects were misclassified for vitamin C. Among 58 subjects who were misclassified into the lower quartile by estimation from dietary intake alone compared with that based on dietary intake plus non-dietary intake for vitamin C, 55 subjects (95%) were classified into the highest intake category by estimation based on dietary intake plus non-dietary intake.

**Table 4.** Distribution of vitamin C intake estimation by quartiles of dietary intake alone and dietary plus non-dietary intake in the Tsurugaya Project (n=1168),* Japan, 2002.

Quartiles of dietary intake alone	Intake (mg)		Quartiles of the estimate from dietary intake + Non-dietary intake(mg)^†^				Total

			1 (highest)	2	3	4 (lowest)	
			≥136.30	104.15 – 136.29	75.50 – 104.14	<75.50	
1 (highest)	≥129.55	n(%)	237 (81.2)	55 (18.8)	0 (0.0)	0 (0.0)	292 (100)
2	100.39 – 129.54	n(%)	23 (7.9)	236 (80.8)	33 (11.3)	0 (0.0)	292 (100)
3	74.38 – 100.38	n(%)	19 (6.5)	0 (0.0)	257 (88.0)	16 (5.5)	292 (100)
4 (lowest)	<74.38	n(%)	13 (4.4)	1 (0.3)	2 (0.7)	276 (94.5)	292 (100)

Total			292 (100)	292 (100)	292 (100)	292 (100)	1168 (100)

* : Subjects were grouped into quartiles according to their dietary intake and their dietary plus non-dietary intake.

† : Non-dietary nutrient sources included vitamin supplement, over the counter drugs, and prescription drugs.

For vitamin E, among the 292 subjects who were classified as quartile 1 on the basis of the estimate from dietary intake plus non-dietary nutrient intake, 34.3 % were misclassified as quartile 2, 3, or 4 in the estimate from dietary intake alone (Table 5). Among the subjects classified as quartile 2 in the estimate from dietary intake plus non-dietary nutrient intake, 34.2 % were misclassified as quartile 1 in the estimate from dietary intake alone. Similarly, 22.3 % in quartile 3 and 9.6 % in quartile 4 were misclassified (Table 5). The overall degree of agreement between these two classifications was 74.9 % for vitamin E. Thus, overall, 25.1 % subjects were misclassified for vitamin E.

**Table 5.** Distribution of vitamin E intake estimation by quartiles of dietary intake alone and dietary plus non-dietary intake in the Tsurugaya Project (n=1168),* Japan, 2002.

Quartiles of dietary intake alone	Intake (mg *α*-TE)^‡^		Quartiles of the estimate from dietary intake + Non-dietary intake(mg *α*-TE)^†‡^				Total

			1 (highest)	2	3	4 (lowest)	
			≥7.503	6.006 – 7.502	4.690 – 6.005	<4.690	
1 (highest)	≥6.918	n(%)	192 (65.8)	100 (34.2)	0 (0.0)	0 (0.0)	292 (100)
2	5.752 – 6.917	n(%)	35 (12.0)	192 (65.8)	65 (22.3)	0 (0.0)	292 (100)
3	4.582 – 5.751	n(%)	37 (12.7)	0 (0.0)	227 (77.7)	28 (9.6)	292 (100)
4 (lowest)	<4.582	n(%)	28 (9.6)	0 (0.0)	0 (0.0)	264 (90.4)	292 (100)

Total			292 (100)	292 (100)	292 (100)	292 (100)	1168 (100)

* : Subjects were grouped into quartiles according to their dietary intake and their dietary plus non-dietary intake.

† : Non-dietary nutrient sources included vitamin supplement, over the counter drugs, and prescription drugs.

‡ : alpha-tocopherol equivalent

The 84 users of non-dietary calcium comprised 14 men and 70 women. We therefore analyzed men and women separately. In men, among the 121 subjects who were classified as quartile 1 on the basis of the estimate from dietary intake plus non-dietary nutrient intake, 5.0% were misclassified as quartile 2 or 3 in the estimate based on dietary intake alone (Table 6). In women, the corresponding figure was 7.0% (Table 7). The overall degree of agreement between these two classifications was 95.9% for men and 87.1% for women. In women, among 41 subjects who were misclassified into the lower quartile by estimation from dietary intake alone compared with that from dietary intake plus non-dietary intake for calcium, 12 (29%) were classified into the highest intake category by estimation from dietary intake plus non-dietary intake.

**Table 6.** Distribution of calcium intake estimation by quartiles of dietary intake alone and dietary plus non-dietary intake in the Tsurugaya Project in men (n=487),* Japan, 2002.

Quartiles of dietary intake alone	Intake (mg)		Quartiles of the estimate from dietary intake + Non-dietary intake(mg)^†^				Total

			1 (highest)	2	3	4 (lowest)	
			≥758.87	623.10 – 758.86	489.20 – 623.10	<489.20	
1 (highest)	≥749.40	n(%)	115 (95.0)	6 (4.9)	0 (0.0)	0 (0.0)	121 (100)
2	620.44 – 749.39	n(%)	2 (1.7)	116 (95.1)	4 (3.3)	0 (0.0)	122 (100)
3	484.10 – 620.43	n(%)	4 (3.3)	0 (0.0)	116 (95.1)	2 (1.6)	122 (100)
4 (lowest)	<484.10	n(%)	0 (0.0)	0 (0.0)	2 (1.6)	120 (98.4)	122 (100)

Total			121 (100)	122 (100)	122 (100)	122 (100)	487 (100)

* : Subjects were grouped into quartiles according to their dietary intake and their dietary plus non-dietary intake.

† : Non-dietary nutrient sources included calcium supplement, over the counter drugs, and prescription drugs.

**Table 7.** Distribution of calcium intake estimation by quartiles of dietary intake alone and dietary plus non-dietary intake in the Tsurugaya Project in women (n=681),* Japan, 2002.

Quartiles of dietary intake alone	Intake (mg)		Quartiles of the estimate from dietary intake + Non-dietary intake (mg)^†^				Total

			1 (highest)	2	3	4 (lowest)	
			≥820.63	666.72 – 820.62	525.75 – 666.71	<525.75	
1 (highest)	≥806.49	n(%)	159 (93.0)	12 (7.0)	0 (0.0)	0 (0.0)	171 (100)
2	650.93 – 806.48	n(%)	9 (5.3)	139 (81.8)	22 (12.9)	0 (0.0)	170 (100)
3	508.18 – 650.92	n(%)	2 (1.1)	17 (10.0)	138 (81.2)	13 (7.6)	170 (100)
4 (lowest)	<508.18	n(%)	1 (0.6)	2 (1.1)	10 (5.9)	157 (92.4)	170 (100)

Total			171 (100)	170 (100)	170 (100)	170 (100)	681 (100)

* : Subjects were grouped into quartiles according to their dietary intake and their dietary plus non-dietary intake.

† : Non-dietary nutrient sources included calcium supplement, over the counter drugs, and prescription drugs.

Among the subjects who were classified as quartile 1 on the basis of the estimate from dietary intake plus non-dietary nutrient intake, vitamin E had the highest degree of misclassification (34.2%), followed in order by vitamin B_1_ (28.8%), vitamin C (18.8%), and calcium (7.0 %) in women, and calcium (5.0%) in men, as quartile 2, 3, or 4 in the estimate based on dietary intake alone.

## DISCUSSION

Our findings show that nutrient intake estimation without consideration of the non-dietary nutrient intake may result in up to one-third of subjects being misclassified. Similar result was shown in a study of Messerer et al.^13^ The misclassification of classified subjects in the highest quintile were 41% when α-tocopherol supplement source was included.^13^ This misclassification may greatly influence the results of epidemiologic studies of the relationship between dietary factors and health outcomes. In contrast to vitamins, our data suggest that the degree of misclassification for calcium may be relatively small.

According to the results of The National Nutrition Survey in Japan,^10^ the mean nutrient intakes for individuals aged over 70 years were 0.78 mg (SD, 0.36) for vitamin B_1_, 131 mg (SD, 90) for vitamin C, 7.7 mg (SD, 4.0) for vitamin E, and 540 mg (SD, 284) for calcium. Thus, the mean intakes of vitamin B_1_, vitamin C, vitamin E, and calcium among our subjects were quite consistent with the mean values in Japan.

In this study, non-dietary nutrient intake was considerably higher than dietary intake alone, especially, vitamin B_1_, vitamin C and vitamin E (Figures 1-3) because of the OTC drug or prescription drug. The daily dosage of OTC drug or prescription drug was higher than supplement intake. For example, the daily dosage of prescription drug was 100mg for vitamin B_1_, 2000mg for vitamin C, 483mg for vitamin E. Several subjects had taken the OTC drug or prescription drug with vitamin supplement simultaneously.

Our study had several methodological strengths. First, the sample was drawn from a Japanese community-dwelling population, thereby minimizing any selection bias associated with clinical samples. Second, our study had a reasonably large sample size, which gave us the opportunity to base our estimate of the degree of misclassification on an adequate sample size. Third, our non-dietary nutrient intake data collections were highly reliable because almost all subjects in our study brought almost all vitamin and mineral supplements, OTC drugs, and prescription drugs that they were taking regularly to the examination site, and one trained pharmacist transcribed the name, content, daily dosage and manufacturer’s name from the label on the container. Only 10 of the 1,178 subjects did not bring their non-dietary nutrients to the examination site. If more subjects had been taking non-dietary nutrients regularly, and did not report or bring their non-dietary nutrients to the examination site for any reason, then the true proportions of misclassification might have been larger than the observed proportions.

Our study also had some limitations. First, most of the subjects were active and healthy enough to participate in the survey, and this might have led to small inter-individual differences in the study data. Therefore, we have likely underestimated the degree of misclassification. Second, we obtained data only for persons aged 70 years or older. Thus, it is uncertain whether our finding of misclassification would also be observed in middle-aged persons, who may tend not to take prescribed vitamin or mineral drugs that elderly individuals take. Finally, we based our calculation of non-dietary nutrient intake on the assumption that our subjects followed the recommendations on the label of the container or daily dosage for OTC drugs and prescription drugs. Some subjects might have taken the supplements regularly, but sometimes compliance with recommendations might be poor. If we had had enough time to ask our subjects about detailed usage of non-dietary nutrient intake in addition to photocopying the label of the container, our method of estimating non-dietary nutrient intake might have been more appropriate than a personal interview without information from the containers, or a self-administered questionnaire.^20^

Block et al^12^ examined data from the NCI-USDA Vitamin C Study and showed that the intake of vitamin C from food plus vitamin C supplement was reflected by the plasma concentration of ascorbic acid. However, when vitamin C intake from food alone was examined, the plasma concentration of ascorbic acid did not reflect the intake. Thus, the exclusion of the vitamin C supplement intake had a great impact in estimation of nutrient intake. Although Block et al^12^ did not examine the extent of the misclassification in nutrient intake estimation in their study, their findings along with our findings strongly suggest that the intake of non-dietary vitamins and calcium should be included in epidemiologic studies investigating the role of nutrients on health.

We examined epidemiologic studies concerning the influence of the dietary intake including vitamin on health outcomes in the original articles published in The Journal of Epidemiology from 2003 through 2005. There were four original articles related to the effects of the dietary intake including vitamin intake on health outcomes.^21^^-^^24^ These studies did not include the intake of vitamin supplements in their analyses or did not mention supplements. Because these studies were started before 1999, the use of supplements at that time might not have been as common as it is currently. However, if these studies had considered the intake of supplements at the baseline or during follow-up, then their conclusions might have differed.

In conclusion, our data suggest that nutrient estimation without consideration of intake from non-dietary sources would greatly affect the results of studies of the relationship between nutrients and health outcomes. We believe that information regarding the intake of vitamins and calcium from vitamin and mineral supplements, OTC drugs, and prescription drugs, should be obtained from subjects by an accurate method when the nutrient intake is estimated in epidemiologic studies.
